# Illustration of charge transfer in graphene-coated hexagonal ZnO photocatalysts using Kelvin probe force microscopy†

**DOI:** 10.1039/c7ra12037k

**Published:** 2018-01-03

**Authors:** Yunlong Zhang, Yuzhi Zhang, Lixin Song, Yang Su, Yunfeng Guo, Lingnan Wu, Tao Zhang

**Affiliations:** The Key Laboratory of Inorganic Coating Materials, Shanghai Institute of Ceramics, Chinese Academy of Sciences 1295 Dingxi Road Shanghai 200050 China lxsong@mail.sic.ac.cn yzzhang@mail.sic.ac.cn; University of Chinese Academy of Sciences Beijing 100049 China; School of Materials Science and Engineering, Shanghai University No. 99 Shangda Road Shanghai 200444 China

## Abstract

A graphene coated hexagonal ZnO (HZO@Gr) with enhanced activity in photocatalysis was synthesized. However, the photoinduced charge transfer behavior and the beneficial role of graphene in promoting photocatalytic reactions have not been sufficiently investigated experimentally. In this paper, the surface potentials of the ±(0001)-polar plane of HZO (Zn-polar plane and O-polar plane), graphene, graphene/Zn-polar plane and graphene/O-polar plane were measured using Kelvin probe force microscopy (KPFM). On the basis of the KPFM results, the respective Fermi levels were calculated and the internal electric field (IEF) of HZO was confirmed. Taking the IEF of HZO into consideration, the three-dimensional band diagrams of the HZO@Gr composites in methyl blue (MB) solution in the dark and under UV-visible irradiation after equilibrium were proposed. Accordingly, it is found that there could emerge different interactions between graphene and HZO at the ±(0001)-polar plane of HZO. Furthermore, the photogenerated holes and electrons tend to migrate to opposite directions. With the participation of graphene and IEF, the composites show a decrease in possibility of charge recombination. As a result, the active groups, namely ˙OH and ˙O_2_^−^ radicals, could be mainly generated at/near the O-polar plane and Zn-polar plane, respectively. This work can serve as a supplemental explanation of the charge transfer during the photocatalytic process at the polar ZnO/graphene composite surface.

## Abstract 2

The Fermi levels and three-dimensional band diagrams of the synthesized HZO@Gr composites in methyl blue (MB) solution before and after equilibrium were assumed.

## Graphical abstract

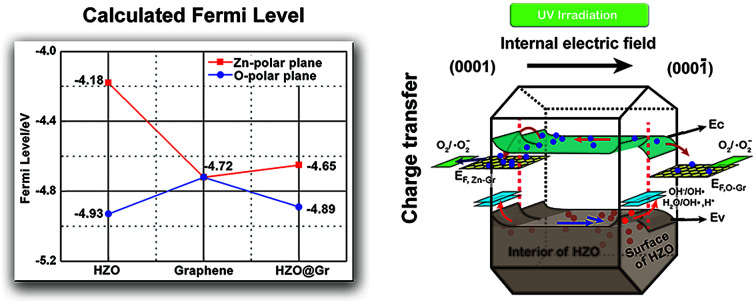

## 1. Introduction

Photocatalysis is a light induced catalytic process that can effectively degrade organic molecules in the water through oxidation and reduction reactions. As one of the most important semiconductor photocatalysts, ZnO has attracted considerable interest because of its high photosensitivity and stability.^1,2^ However, despite its great potential, the photocatalytic efficiency remains very low because of the wide band gap and the fast recombination of the photogenerated electron–hole pairs.^3,4^ There are several methods to enhance the photocatalytic efficiency of ZnO. It is found that an increase of polar Zn (0001) or O (0001̄) facets leads to a significant enhancement of photocatalytic activity, whereas the area of nonpolar (*i.e.*, 011̄0, 101̄0) planes has negligible influence on the photocatalysis.^5,6^ The periodic density functional theory (DFT) demonstrates each layer of HZO contains all positive Zn^2+^ ions or all negative O^2−^ ions in the [0001] direction and an internal electric field (IEF) thus is generated between Zn–HZO (0001) and O–HZO (0001̄) planes due to the spontaneous polarization.^7^ When HZO particles are irradiated by UV-visible light, photogenerated electrons (e^−^) and holes (h^+^) migrate to positive polar Zn–HZO (0001) planes and negative polar O–HZO (0001̄) planes, respectively, under the IEF, thus promoting the separation of photogenerated electron–hole pairs.^8,9^ In addition, since the graphene was coming into a hot topic of researching, the graphene–semiconductor composites have attracted increasing interests for researchers so that to improve the efficiency of the photocatalytic processes.^10–13^ Hence, the photocatalytic performance of ZnO can be obviously enhanced by combining with graphene, which possesses a unique sp^2^ hybrid carbon network and excellent mobility of charge carriers at room temperature.^14^ It is reported that photogenerated electrons tend to transfer to graphene for further charge separation, which could obviously decrease the recombination possibility.^15–17^

The transfer behavior of electrons at the interfaces of composite is determined by the work function of respective species. Although the photocatalysis mechanism of ZnO/graphene composites has been explained in detail, and the existence of IEF for hexagonal structure ZnO has been demonstrated by theoretical calculations, the photocatalysis mechanism of ZnO/graphene still needs to be further analyzed by characterizing surface electrical properties with the help of more powerful experiment. The atomic force microscope (AFM) based Kelvin probe force microscopy (KPFM) technique has already been demonstrated as a powerful tool for measuring electrostatic forces and electric potential distribution with nanometer resolution. The technique has been applied to materials science applications such as: work function mapping,^18^ characterization of dopant profile of semiconductors in a nanometer resolution,^19^*etc.* Lin *et al.*^20^ have studied the charge transfer in Au nanoparticle–nonpolar ZnO photocatalysts by measuring the surface potential of the composites using KPFM, thus obtaining the surface-potential-derived band diagram, which exhibited a particular method to study the charge transfer of the photocatalysts in the photocatalysis process.

Although the mechanism that the combination of graphene can enhance the photocatalytic efficiency has been proposed theoretically, there are still lack of experimental supports. To explore the transfer behaviors of photogenerated charges before and after combination of graphene, and the degradation mechanism of MB under UV-visible light, the KPFM method was applied in this paper. Different surface potential of Zn-polar planes and O-polar planes was found and thus the IEF of HZO was estimated. Surface potential of Zn-polar plane, O-polar plane, graphene, Gr/Zn-polar plane and Gr/O-polar plane was measured. Accordingly, the relevant Fermi levels were calculated and the band diagrams of interfaces, namely Gr/Zn-polar plane and Gr/O-polar plane in the dark and under UV-visible irradiation were proposed. According to the energy potential of the corresponding species of MB solution reported in literatures, the band diagrams of HZO/MB_(aq)_ and HZO@Gr/MB_(aq)_ interfaces were suggested in the dark and under UV-visible irradiation. On the basis of the obtained conclusions, the three-dimensional band diagrams of HZO/Gr, HZO/MB_(aq)_ and HZO@Gr/MB_(aq)_ interfaces were proposed for a comprehensive understanding of the charge transfer in the HZO–MB_(aq)_, HZO@Gr–MB_(aq)_ systems when photodegradation process took place. The results show that Zn-polar planes and O-polar planes could undertake different degradation reactions under IEF.

## 2. KPFM measurement method

KPFM is similar to noncontact-mode AFM. It measures electrostatic force in addition to atomic force. By measuring the contact potential difference (CPD) which minimizes the electrostatic force between probe tip and sample surface, the work function difference between tip and surface is estimated. The CPD between probe tip and sample is defined as^21^1
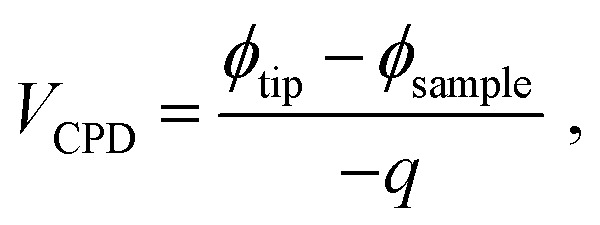
where *ϕ*_tip_ and *ϕ*_sample_ are the work function of the tip and the sample, respectively, and *q* is the elementary charge. If the work function of selected probe tip is constant, the difference of the work function between two samples is the negatively numeric equivalent of the difference between CPD. Therefore, by loading the samples onto a substrate whose work function is known, we can estimate the work function of the samples, thus referring the location of relevant Fermi levels.

## 3. Experimental and characterization procedures

### 3.1 Preparation of HZO

The polar-plane dominant HZO was synthesized using a facile method. First, 75 ml deionized water and 75 ml *N*-methyl-2-pyrrolidone (NMP) were mixed in a conical breaker. Then 4.0 g zinc acetate dehydrate (Zn(Ac)_2_·2H_2_O) was added into the breaker. At the same time, the mixed solution was stirred continually and heated to 95 °C. After 5 hours, the acquired turbid solution was filtrated and the precipitate was washed with water at least 3 times to remove the remaining NMP. Finally, the product was drying at 80 °C for 6 hours to get the HZO powder.

### 3.2 Synthesis of graphene-coated HZO (HZO@Gr)

Generally, the HZO@Gr composite was obtained by self-assembly of the graphene oxide (GO) and amine-functionalized HZO (HZO-NH_2_). First, GO was synthesized using the modified Hummer's method, which was reported elsewhere.^22^ The exfoliated GO was dispersed in deionized water and centrifuged at 8500 rpm for 15 min. Then the supernatant was diluted to the concentration of 0.1 mg ml^−1^ for storage. The HZO surface was amine-functionalized by mixing with 2.5% amino-propyl-trimethoxysilane (APTMS)/ethanol solution and meanwhile stirring for 8 hours at 70 °C. Then the turbid solution was washed with ethanol 3 times and the obtained precipitants were dried at 80 °C for 3 hours. Subsequently, 0.5 g acquired HZO-NH_2_ was dispersed in 70 ml deionized water by ultrasonic dispersion and then mixed with 8 ml GO solution mentioned above. The mixed solution was magnetically-stirred at 55 °C for 8 hours. After that, the turbid solution was washed 3 times with water by centrifugation to get the precipitate and subsequently drying for 5 hours at 80 °C. Finally, the obtained HZO@GO powder was thermal-reduced in Ar for 2 hours at 450 °C to get the HZO@Gr composite.

### 3.3 Characterizations

The microstructures of HZO and HZO@Gr were characterized by scanning electron microscopy (SEM, Hitachi S-3400N) equipped with energy-dispersive spectrometry (EDS, XFlash Detector 5010, Bruker, Germany), field emission SEM (FESEM, Magellan 400, FEI Company, USA) and high-resolution transmission electron microscope (HRTEM, Tecnai G2 F20, FEI Company, USA). The KPFM measurements were conducted with atomic force microscope (AFM, Dimension Icon, Bruker, Germany). UV-visible absorption spectra were measured using a UV-Vis-NIR spectrophotometer (CARY 5000 Scan, Varian, USA).

### 3.4 Evaluation of photocatalytic activity

The photodegradation process of methyl blue (MB) was tracked based on the absorption spectroscopic technique. 50 mg samples were dispersed in 100 ml MB aqueous solution (10 mg l^−1^). The mixed suspensions were magnetically stirred for 1 h under dark in ambient conditions to get the absorption–desorption equilibrium before irradiation. Then the suspensions were exposed to the light irradiation produced by a 300 W Xe lamp positioned at a 36 cm distance away from the vessel. At certain time intervals, 3 ml of the mixed suspensions were extracted and centrifuged to remove the photocatalyst. The degradation MB solution was analyzed by recording changes of the absorption at the maximum band using a UV-vis absorption spectrometer.

### 3.5 KPFM measurement

KPFM measurements were performed in lift mode in which the surface would be scanned for two times in each line of the sample during one test procedure to make sure the topography and CPD images were acquired in the same positions. Namely, the forward line was scanning to record the topography of the surface and the backward line was scanning at a chosen lift height along the same line so as to measure the CPD between probe tip and the surface of sample. A conductive probes (Bruker, SCM-PIT-V2) with silicon cantilevers coated with PtIr and Sb with a nominal resonance frequency of 75 kHz were used in this experiment. The substrate, namely highly oriented pyrolytic graphite (HOPG) was used as reference sample. The graphene sample was obtained by spin-coating diluted GO solution onto HOPG at 500 rpm and subsequent annealing in Ar at 450 °C for 2 h. HZO and HZO@Gr were dispersed in ethanol and spin coated onto HOPG at 500 rpm to acquire HZO and HZO@Gr samples.

## 4. Results and discussion

### 4.1 Morphology of HZO@Gr composite

**Fig. 1:**
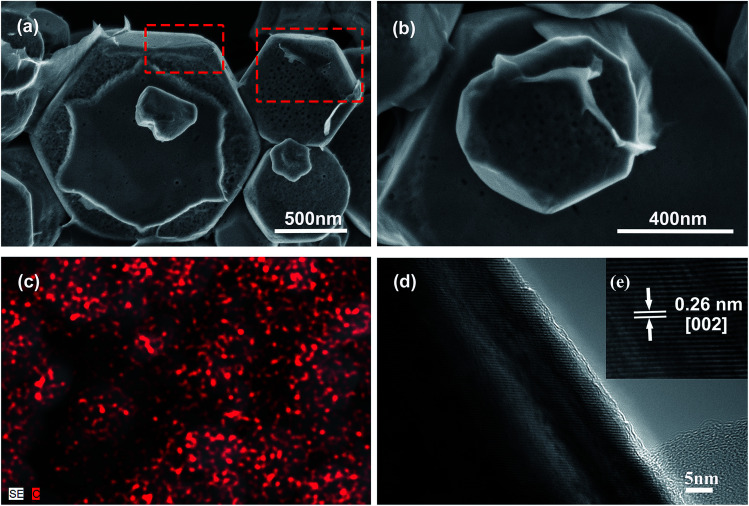
(a, b) TEM images of HZO@Gr. (c) Carbon elemental mapping analysis image of HZO@Gr. (d) HRTEM image of HZO@Gr. (e) Magnified image of (d), revealing that the distance between two (002) planes of the hexagonal ZnO phase with the interplanar spacing in the crystalline petal is 0.26 nm.

Fig. 1a and b show the FESEM images of HZO@Gr composite. The obtained HZO particles possess regular hexagonal shape. We can see the upturned layers of graphene on the surface of HZO particle in Fig. 1a. The areas marked by the red dotted line exhibit that there exists color or height difference when graphene covers on the HZO surface so that we can see the boundary of graphene. Meanwhile, the surface of HZO particle is covered by transparent graphene film, which seems that it is wrapped by some transparent plastic film and the wrinkle can also be seen (Fig. 1b). The detailed combination state of graphene and HZO can be seen from HRTEM in Fig. 1d. There are about 3 layers of graphene at the edge zone of HZO, indicating that HZO are well coated by graphene. In addition, the magnification of Fig. 1d (Fig. 1e) shows that the interplanar spacing in the crystalline petal is 0.26 nm, which corresponds to the distance between two (002) planes of the hexagonal ZnO phase, indicating preferential growth along the [002] direction. Furthermore, the carbon elemental mapping analysis image in Fig. 1c also exhibited plenty of red dots densely covering on the HZO particles, indicating the surface of HZO particles are coated by graphene.

The mechanism for the formation of HZO@Gr was demonstrated in our previous work,^23^ which was based on the electrostatic-force-induced self-assembly process. In brief, the surface of HZO was amine-functionalized by APTMS as the eqn (2) to form a positive-charged surface. The strongly negatively groups (–COOH, –O–, –OH) on GO make the GO negatively charged. The difference of surface potential between HZO and GO could cause enough electrostatic force so as to attract them together and form a well graphene-coated structure.2
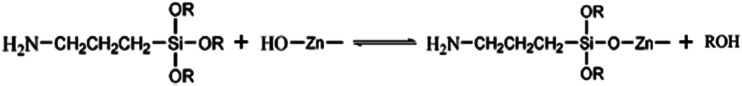


### 4.2 Photocatalytic property

**Fig. 2:**
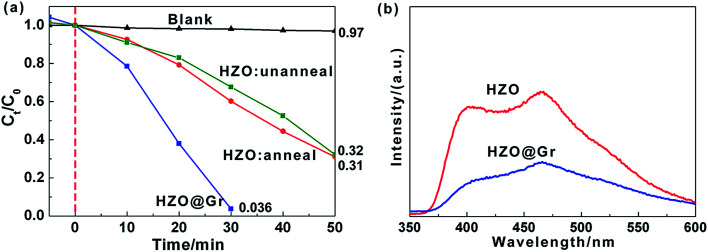
(a) Photodegradation of MB over HZO@Gr, HZO (anneal), HZO (unanneal) and blank, respectively. (b) The room temperature PL emission of HZO and HZO@Gr.

The photocatalytic experiments were carried out in order to study the influence of graphene on photocatalytic properties of as-prepared HZO. Fig. 2a shows the results of the MB degradation with different photocatalysts, where *C*_0_ and *C*_*t*_ are the initial concentration after the equilibrium adsorption and the residual concentration of MB, respectively. The concentration of MB at each period is correlated with the maximum absorption peaks of MB in the UV-visible spectra, which were shown in ESI (Fig. S1†). The results indicate that for the degradation of MB, it shows negligible photocatalytic efficiency without catalyst, while the catalytic rate of MB by HZO (unanneal) enhances obviously with 0.61 and 0.31 of MB remained after 30 min and 50 min of irradiation, respectively. What's more, the residual MB after 50 min degradation is 0.32 for HZO annealed and 0.31 unannealed, which indicates the calcination process almost has no effects on photodegradation of MB. The possible reason might be that the anneal temperature of 450 °C is relative low compared with the melting point of ZnO (1975 °C) and has little effect on HZO structure. So we can regard the calcination process almost has no effect on photocatalysis efficiency. In contract, the residual MB reached to 0.036 after 30 min irradiation in the presence of HZO@Gr, which indicated that the coating of graphene produces some interaction between graphene and the surface of HZO, thus affecting the surface electron behavior of HZO under the UV-visible light irradiation. The photoluminescence spectra of HZO and HZO@Gr (Fig. 2b) show that HZO@Gr exhibits much weaker emission at 395 and 465 nm than HZO, which result from band-edge emission caused by the recombination of excitonic centers^24,25^ and bound excitons caused by the intrinsic defects.^26,27^ These may be explained by two possible mechanisms: (1) the introduction of graphene can eliminate the surface defects, and (2) the graphene can accept the photo-induced electrons assuredly and improve the charge separation.^27^

**Fig. 3:**
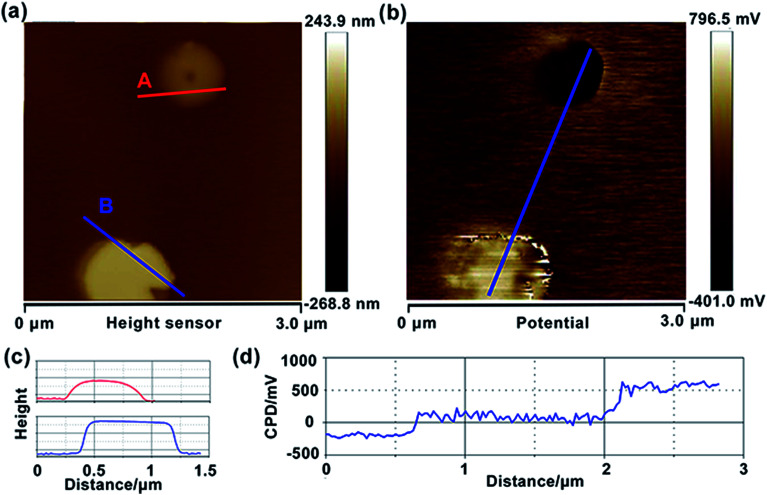
Topography (a) and CPD (b) images of HZO particles, deposited on HOPG. Topography (c) and CPD cross-section (d) analysis of HZO.

To explore the photocatalytic mechanism of HZO@Gr, the AFM topography and KPFM CPD (surface potential) of HZO, graphene and HZO@Gr were measured. Fig. 3a was the AFM topography of HZO particle A and B with the thickness of 75.7 and 145.7 nm, respectively. The KPFM measurement profiles of CPD in Fig. 3b exhibit two opposite potential on the surface of A and B, which are 556.1 and −194.7 mV, respectively. It is known that atomic stacking of the wurtzite ZnO polar crystal in which O^2−^ and Zn^2+^ ions stack alternatively along the *c*-axis results in positive charged Zn-terminated (0001) and negative charged O-terminated (0001̄) planes.^8,28^ An IEF is thus generated between the two polar planes due to the spontaneous polarization.^7^ Because it is difficult to measure both surface potential of a HZO particle, we consider the difference between the *V*_CPD_ is the potential difference of the HZO particle, thus the IEF of HZO is3
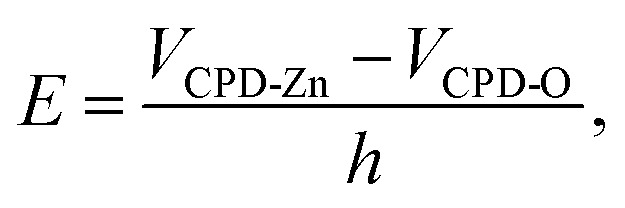
where *V*_CPD-Zn_ and *V*_CPD-O_ are surface potential of Zn-polar plane and O-polar plane, *h* stands for the thickness of HZO particle. Hence, the IEF of particle A and B are estimated 9.9 × 10^4^ and 5.2 × 10^4^ V cm^−1^. For the high electron mobility of ZnO, it is reasonable to believe numerous photogenerated electrons and holes will be driven to [0001] and [0001̄] direction. On the other hand, the work function of Zn-(0001) and O-(0001̄) planes can be calculated by the measured CPD of each plane. What's more, the work function of HOPG in air is regarded as 4.65 eV.^29^ By taking the average value of respective potential as the CPD between tip and the surface of the sample, the work function of Zn-(0001) and O-(0001̄) was calculated to be 4.18 and 4.93 eV, which are approximated with the reported value (4.25 and 4.95 eV).^30^ Since the work function is correlated with Fermi level of each surface, when taking the vacuum level as 0 eV in this article, the Fermi level of Zn-(0001) and O-(0001̄) polar planes are −4.18 and −4.93 eV.

**Fig. 4:**
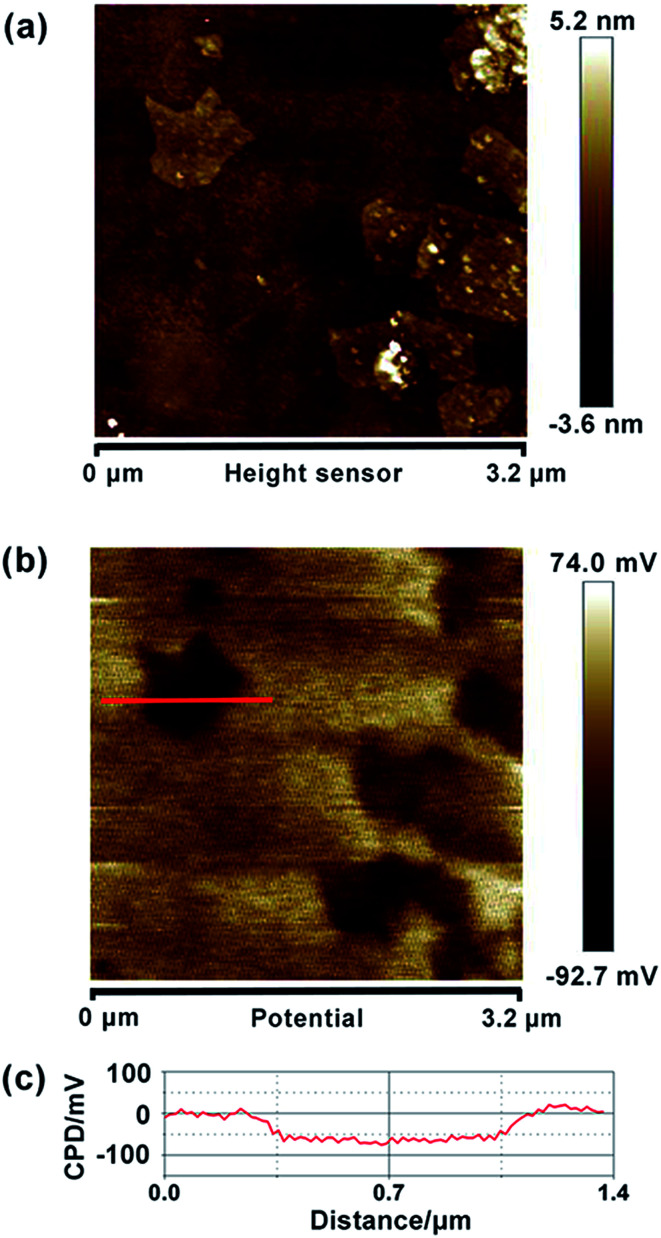
Topography (a) and CPD (b) images of graphene particles, deposited on HOPG. (c) CPD cross-section of graphene.

**Fig. 5:**
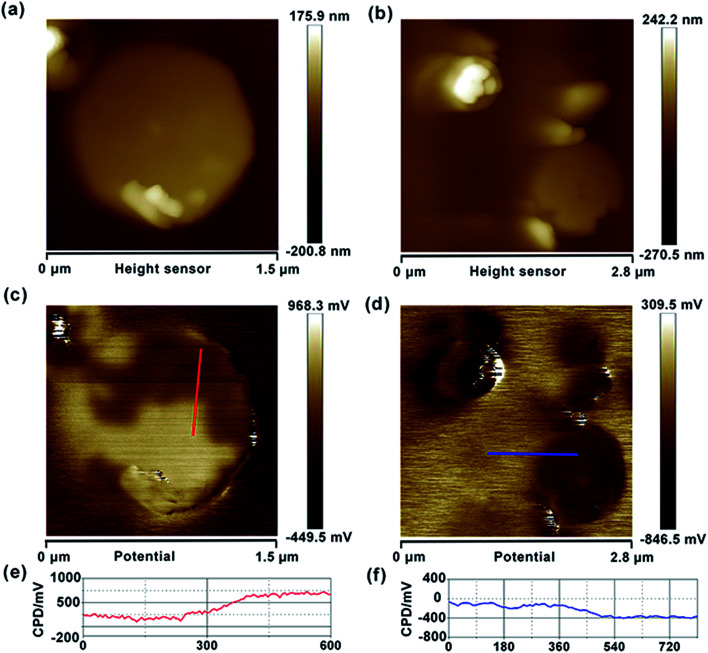
Topography images of Gr/Zn-polar plane (a) and Gr/O-polar plane (b). CPD images of Gr/Zn-polar plane (c) and Gr/O-polar plane (d). CPD cross-section of Gr/Zn-polar plane (e) and Gr/O-polar plane (f).

**Fig. 6:**
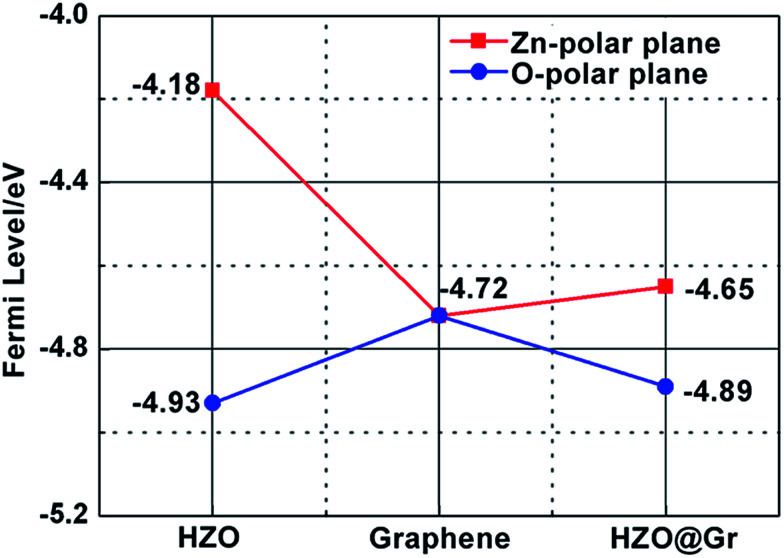
The calculated Fermi levels of HZO, graphene and HZO@Gr. The red line stands for Zn-polar plane and blue line represents O-polar plane.

Similarly, we also measured the AFM topography and CPD images of the obtained graphene, HZO@Gr, which are shown in Fig. 4 and 5. The size of graphene sheets is approximately several hundred nanometers so as to better cover the surface of HZO particles. The work function of graphene was calculated to be 4.65 eV, which is a little larger than pristine graphene reported by others.^31^ It might be caused by the remaining negatively charged oxygen-containing groups or the destroy of the structure during annealing that form the localization of the sp^2^ area and decrease the density of free electron on the whole graphene sheet. The KPFM images of Zn-(0001) and O-(0001̄) plane of HZO are showed in Fig. 5a and b. It is noteworthy that the Zn-(0001) surface exhibits two different surface potential, which may arise from the combination of graphene sheets. If the work function of Zn-(0001) and O-(0001̄) polar planes are regarded as 4.18 and 4.93 eV mentioned above, the work function of the graphene on Zn-(0001) and O-(0001̄) polar planes were calculated to be 4.66 and 4.87 eV, respectively. For the calculated Fermi levels are located between graphene and HZO surface and the low layer number of graphene, we can regard the calculated Fermi levels are corresponding to the work functions. Hence, the Fermi levels of graphene (*E*_F,Gr_), Zn-polar plane (*E*_F,Zn_), O-polar plane (*E*_F,O_), Zn–graphene surface (*E*_F,Zn–Gr_), O–graphene surface (*E*_F,O–Gr_) were acquired (Fig. 6).

**Fig. 7:**
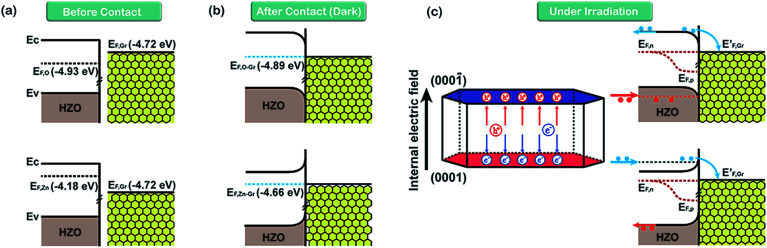
The inferred band diagrams of HZO@Gr composite based on calculated Fermi levels in Fig. 6. (a) Before O- and Zn-polar planes make contact with graphene, (b) after contact with MB in the dark and (c) under UV-visible irradiation. *E*_F,O_ and *E*_F,Zn_ are the Fermi level of O- and Zn-polar plane. *E*_F,O–Gr_ and *E*_F,Zn–Gr_ represent the Fermi level of Gr/O-polar plane and Gr/Zn-polar plane.

On the basis of the measured Fermi levels of HZO, graphene and HZO@Gr, the proposed band diagram of HZO and graphene in the dark before contact formation is sketched in Fig. 7a. The Fermi levels of O- and Zn-polar plane are lower and higher than that of graphene respectively, indicating the electrons transfer from graphene to O-polar plane and from Zn-polar plane to graphene between the two interfaces after contact formation in order to align the Fermi levels. Similar to the Au–ZnO composites,^32^ a Schottky contact even Ohmic contact is thus established at the junction of O-polar plane and graphene while only Schottky contact can be formed at the junction of Zn-polar plane and graphene for the different bending orientations of energy bands (Fig. 7b). Fig. 7c shows the possible band diagram of HZO@Gr composite under UV-visible irradiation, where *E*_F,n_ and *E*_F,p_ represent the quasi-Fermi level of photogenerated electrons and holes, respectively. With steady-state UV-visible light irradiation on HZO surface, some photoelectron–hole pairs are separated by either a Schottky barrier or an Ohmic contact but some could recombine immediately. For the O-polar plane, once the photogenerated electron–hole pairs were separated, the photoelectrons in the conduction band (CB) of O-polar surface tend to transfer to the Fermi level of graphene due to the as-established Schottky contact even Ohmic contact in the dark. Hence, graphene is serving as electron traps, which is beneficial to charge separation, causing the decreasing of PL intensity. Nevertheless, there exists a Schottky barrier at the interface of Zn-polar plane and graphene, which may hinder some photoelectrons with low energy transferring to the graphene. However, it can still be predicted that most of the photoelectrons could reach the lowest required energy level (*E*′_CB_) and thus transferring to the graphene. On the one hand, the photoelectrons transferring above the *E*′_CB_ from valance band by absorbing demanded light energy could then transfer to the graphene. On the other hand, besides the photoelectrons excited from the surface, most photoelectrons transferring to the surface may be excited from the interior of HZO. Zhan *et al.* have demonstrated that the effective depth of UV-visible-light absorption of ZnO is around 100 nm,^33^ which is corresponding to the thickness of HZO. Since amounts of photoelectrons are generated in the interior of HZO and immediately exposed to the powerful inter electric field, the photogenerated electrons and holes will be accelerated under the electric field to the opposite directions quickly, thus promoting the separation of electron–hole pairs. For the particles of A and B in Fig. 3a, the photoelectrons generated in the interior of HZO will get an extra energy when they are accelerated to the Zn-polar plane under the inter electric field, which is estimated 0.75 eV at most. Thus the photoelectrons at the bottom of CB may reach to the *E*′_CB_ through accelerating and then transfer to the graphene. It can be concluded that most of the photogenerated electrons have the possibility to transfer to graphene and the excited electron–hole pairs can be separated quickly under inter electric field, which will decrease the possibility of recombination of electron–hole pairs. In addition, under the effects of UV-visible-light irradiation and inter electric field, the Zn-polar plane and O-polar plane could change to electron-enriched and hole-enriched plane, leading to different main chemical reactions during the photodegradation process.

**Fig. 8:**
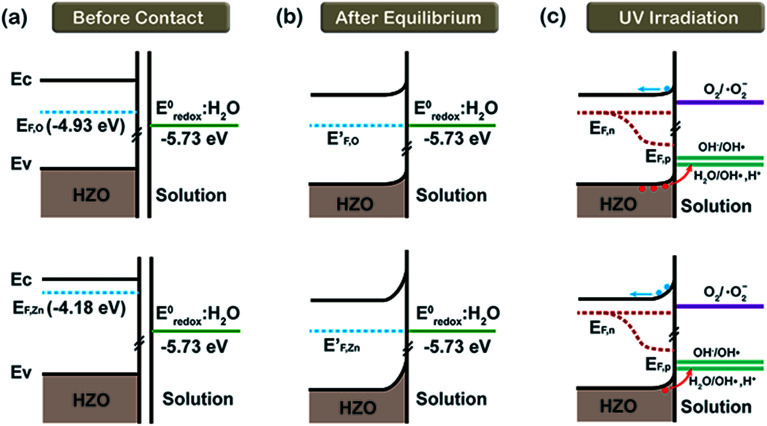
The referred energy diagrams related to the interfaces of MB_(aq)_/O-polar plane and MB_(aq)_/Zn-polar plane. (a) Before contact with MB_(aq)_, (b) after equilibrium formation in MB solution in the dark and (c) under UV-visible irradiation.

On the basis of the conclusions discussed above, it is necessary to discuss the interfaces of HZO–MB_(aq)_ and HZO@Gr–MB_(aq)_ when using HZO@Gr composites as the photocatalysts for the degradation of MB solution. Fig. 8a shows the band diagram related to the HZO–MB_(aq)_ interfaces before they make contact with MB_(aq)_. It can be regard the energy potential of MB_(aq)_ as the standard redox potential of H_2_O (−5.73 eV, *vs.* NHE at 25 °C, which equals −4.5 eV) for the extremely low concentration of MB (10 ppm). When the O-polar plane and Zn-polar plane are contacting with MB solution, there respective Fermi levels tend to align with the corresponding energy level of *E*^0^_redox_:H_2_O until equilibrium is achieved.^16^ As a result, both of the two planes will form a Schottky barrier at the interfaces of HZO and MB_(aq)_, as shown in Fig. 8b. What's more, because the difference between *E*_F,Zn_ and *E*^0^_redox_:H_2_O is larger than *E*_F,O_ and *E*^0^_redox_:H_2_O, the Schottky barrier at the Zn-polar plane will be higher. When UV-visible light illuminates the HZO particles, the electrons in valence band (VB) were excited to CB both in the interior and at the surface of HZO, forming photogenerated electron–hole pairs. The energy levels of OH^−^/˙OH and H_2_O/˙OH, H^+^ shown in Fig. 8c are based on the literature, which are −6.81 eV (ref. 34) and −6.33 eV (ref. 35) (*vs.* NHE at 25 °C). Some of the photogenerated holes migrated to the surface from interior of HZO may react with the adsorbed H_2_O and OH^−^ to form ˙OH radicals to degrade the MB molecules due to the suitability of the energy level for charge transfer. Based on the conclusion that the O-polar plane is abundant in the migrated holes, the main chemical reactions for the O-polar plane are.

4 H_2_O/OH^−^ + h^+^ → ˙OH

While for the electron-enriched Zn-polar plane, the higher Schottky barrier will resist more photogenerated electrons to react with O_2(aq)_, causing the accumulation of electrons in the CB, which may lead to increasing recombination possibility. Taking the energy level of O_2_/˙O_2_^−^ as −4.22 eV (ref. 36) (*vs.* NHE at 25 °C), the electrons with enough energy will transfer to O_2(aq)_ to form the ˙O_2_ radicals which may degrade MB as follows:^15,37^

5 e^−^ + O_2(aq)_ → ˙O_2_^−^

6 ˙O_2_^−^ + H_2_O → H_2_O_2_ + ˙OH

7 H_2_O_2_ → ˙OH

8 ˙OH + MB → CO_2_ + H_2_O

**Fig. 9:**
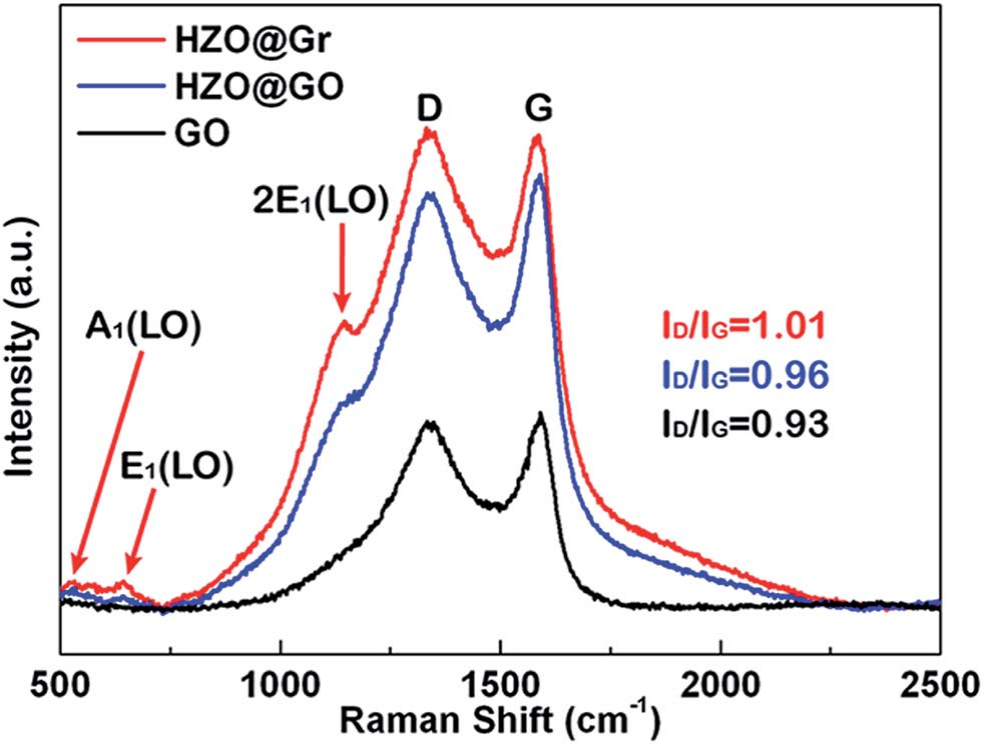
Raman spectra of HZO@Gr, HZO@GO and GO.

**Fig. 10:**
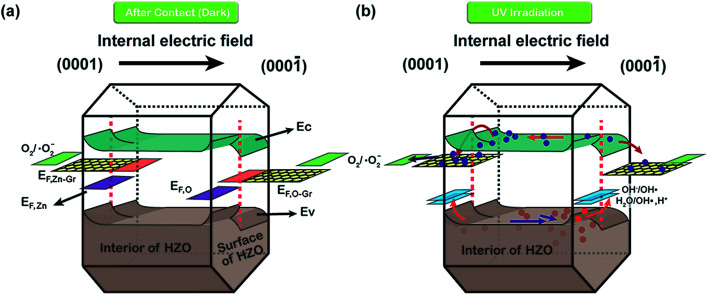
Three-dimensional band diagrams of HZO and HZO@Gr in MB solution (a) in the dark and (b) under UV-visible irradiation. The left side of hexagonal prism stands for Zn-polar plane and the right side represents O-polar plane.

Hence, the produced hydroxyl radicals are the direct strong oxidant to decompose the MB molecule. However, it seems that the photoelectrons will accumulate at the Zn-polar plane of pure HZO particles, which may lead to the high possibility to recombine with the holes. In contrast, the three-dimensional band diagrams of HZO and HZO@Gr composite in the dark and under UV-visible irradiation are shown in Fig. 10a and b. The hexagonal prism diagram represents energy bands of (0001) Zn-polar plane, interior of HZO and (0001̄) O-polar plane, aiming to compare the differences of the energy bands whether combining with graphene. The energy bands in the front part of hexagonal prism are related to bare HZO and the back part referring to HZO@Gr. When HZO is contacting with MB solution in the dark, a Schottky barrier will form at the interface of HZO and MB solution as explained above. However, the graphene avoids direct contact between MB solution and HZO surface, which may have little effect on the energy levels at the interface of graphene and HZO for the abundant free electrons in the outer graphene could buffer the difference between the energy level of *E*^0^_redox_:H_2_O and Fermi level of graphene. Hence, compared with the Schottky barrier between HZO and graphene, there will appear higher Schottky barrier between HZO and MB solution because the larger difference between energy level of *E*^0^_redox_:H_2_O and Fermi level of HZO will cause more electrons transfer to align the energy levels. When irradiated by UV-visible light, the higher Schottky barrier will hinder more electrons in the CB transferring to the energy level of *E*^0^_redox_:H_2_O. While for HZO@Gr, most of the photogenerated electrons have the possibility to transfer to graphene and the excited electron–hole pairs can be separated quickly under inter electric field, which is discussed in Fig. 7c. Therefore, when contacted with MB solution, graphene can prevent the rise of Schottky barrier and served as a sink for electrons, thus decreasing the recombination possibility of photogenerated electron–hole pairs. Accordingly, when UV-visible light irradiates the HZO@Gr composites, the electrons could be excited to CB. For the interfaces of graphene/Zn-polar plane, if not recombined with holes, then the electrons could be driven to graphene under IEF. Theoretically, the Fermi level of the composite graphene at the Zn-polar side is not appropriate for charge transfer between graphene and O_2_/˙O_2_^−^ to form ˙O_2_^−^ radicals. However, the Raman spectra of GO, HZO@GO and HZO@Gr were measured to study the effect of thermal annealing on the quality of graphene (Fig. 9). The sample of GO, HZO@GO and HZO@Gr show two prominent peaks, which are D band and G band, suggesting the structure of graphene is maintained in the composites. Besides, HZO@GO and HZO@Gr also exhibit peaks at 535, 579 and 1146 cm^−1^, which stand for A_1_, E_1_ and 2E_1_ modes of longitudinal optical (LO) phonons, respectively.^38,39^ Generally, the quality of nanostructured carbon-based materials can be reflected by the ratio of the intensities (*I*_D_/*I*_G_) of the two bands D and G. Compared with GO and HZO@GO, HZO@Gr exhibits obviously increased *I*_D_/*I*_G_ ratio, which means after thermal reduction, the oxygen functional groups in GO was removed and the conjugated graphene network (sp^2^) will be re-established.^40^ As a result, the average size of the sp^2^ domains is decreased and the Fermi level of each fragments could be considered localized, which may be gradually lifted due to photoelectrons transferred to graphene and then stored inside the localized graphene. Further, once the localized Fermi level of graphene is higher than the potential of O_2_/˙O_2_^−^, the reaction to generate ˙O_2_^−^ radicals may occur and lead to the degradation of MB_(aq)_ as elaborated in eqn (5)–(8). As for the holes generated at/near the interface of graphene/Zn-polar plane, some may transfer to graphene for the Schottky contact at the surface, and the others could be driven to O-polar plane under IEF. Correspondingly, for the interface of graphene/O-polar plane, the photogenerated electrons could be either trapped in graphene *via* the Schottky or Ohmic contact of the interface or transferring to Zn-polar plane under IEF. As the excited electrons are relatively scarce at/near the O-polar plane, the electrons may be not so enough as to lift the localized Fermi level of graphene over the potential of O_2_/˙O_2_^−^. While compared with the HZO/MB_(aq)_ interface of O-polar plane side, the valence band could emerge a Schottky barrier at the surface of graphene/O-polar plane. The excess holes could also move laterally to the vicinity of the graphene-uncovered HZO surface for its lower potential energy at HZO/MB_(aq)_ interface. The generated holes would transfer to energy levels of OH^−^/˙OH and H_2_O/˙OH, and contribute to the generation of ˙OH radicals and the following degradation of MB. Furthermore, once the holes transferred to graphene, they could easily recombine with the surrounded electrons, which is unfavorable for the generation of ˙OH. Therefore, the exposed O-polar surface of HZO@Gr composites plays a crucial role in the photodegradation of MB solution. However, although bare HZO has lager MB/O-polar plane interfacial area, its photocatalytic activity seems to be lower, which can be ascribed to the inefficient charge separation. The incomplete coverage and breakage or defects of graphene offer large area of exposed HZO in order to provide a channel for the reaction of the migrated holes and MB solution. As a result, by efficiently separating the photogenerated electron–hole pairs under IEF, the HZO@Gr composites show enhanced photocatalytic performance.

## 5. Conclusion

We have synthesized hexagonal ZnO (HZO) and graphene-coated HZO (HZO@Gr) photocatalysts by solution method. The HZO@Gr composite exhibited enhanced photocatalytic performance and low photogenerated electron–hole recombination possibilities. In order to investigate the effect of graphene and IEF on the photocatalytic process of ±(0001)-polar plane, KPFM method was used to characterize the surface potential of ±(0001)-polar plane, graphene, Gr/Zn-polar plane and Gr/O-polar plane. For HZO particle of A and B, the values of IEF were estimated 9.9 × 10^4^ and 5.2 × 10^4^ V cm^−1^. By calculating their respective work function and Fermi levels, the charge transfer behaviors and the readjustment of energy levels in the dark and under UV-visible irradiation were supposed. According to the energy potential of the corresponding species of MB solution, the photodegradation processes of Zn- and O-polar plane were made clear. The electrons and holes can be generated at the surface and in the interior of HZO. Under IEF, the e^−^ and h^+^ were accelerated to gain extra energy and aggregated at/near Zn- and O-polar plane, respectively. For bare HZO, the high Schottky barrier at the MB_(aq)_/Zn-polar plane could hinder most electrons and thus the excess electrons would increase the possibility of charge recombination. Meanwhile the holes could transfer to the energy levels of OH^−^/˙OH and H_2_O/˙OH because of the Schottky contact at the interface of MB_(aq)_/O-polar plane. As to HZO@Gr, most of the photogenerated electrons have the possibility to transfer to graphene under IEF, which ensured the effective photocarriers separation under illumination. Some of the transformed electrons on graphene could possess enough energy to transform O_2(aq)_ to ˙O_2_^−^. The excess holes, which were laterally transferred to graphene-uncovered HZO, produced ˙OH on the surface. Hence, Zn-polar plane and O-polar plane could mainly generate ˙O_2_^−^ and ˙OH radicals respectively, which are the main species to degrade the MB molecules. The results suggest that the IEF and combination of graphene are the factors to the effective separation of photocarriers, which may improve the photocatalytic activity and decide the generation of intermediate radicals at different polar plane.

## Conflicts of interest

There are no conflicts to declare.

## Supplementary Material

RA-008-C7RA12037K-s001
