# Supplementary material for: Illustration of charge transfer in graphene-coated hexagonal ZnO photocatalysts using Kelvin probe force microscopy
Source: RSC Adv. 2018 Jan 3;8(2):885–94. doi: 10.1039/c7ra12037k (PMC9076950; doi:10.1039/c7ra12037k)
Supplement: RA-008-C7RA12037K-s001 [file RA-008-C7RA12037K-s001.pdf]

Electronic Supplementary Material (ESI) for RSC Advances.
This journal is © The Royal Society of Chemistry 2017

Supporting information for:

Illustration of charge transfer in graphene-coated hexagonal ZnO photocatalysts using Kelvin probe force microscope

Yunlong Zhang[a,b], Yuzhi Zhang[a,*], Lixin Song[a,*], Yang Su[b], Yunfeng Guo[a,c], Lingnan Wu[a], Tao Zhang[a]

a The key Laboratory of Inorganic Coating Materials, Shanghai Institute of Ceramics, Chinese Academy of Sciences,1295 Dingxi Road, Shanghai, 200050

b University of Chinese Academy of Sciences, Beijing 100049, China

*Corresponding author. E-mail addresses: lxsong@mail.sic.ac.cn

*Corresponding author. E-mail addresses: yzzhang@mail.sic.ac.cn

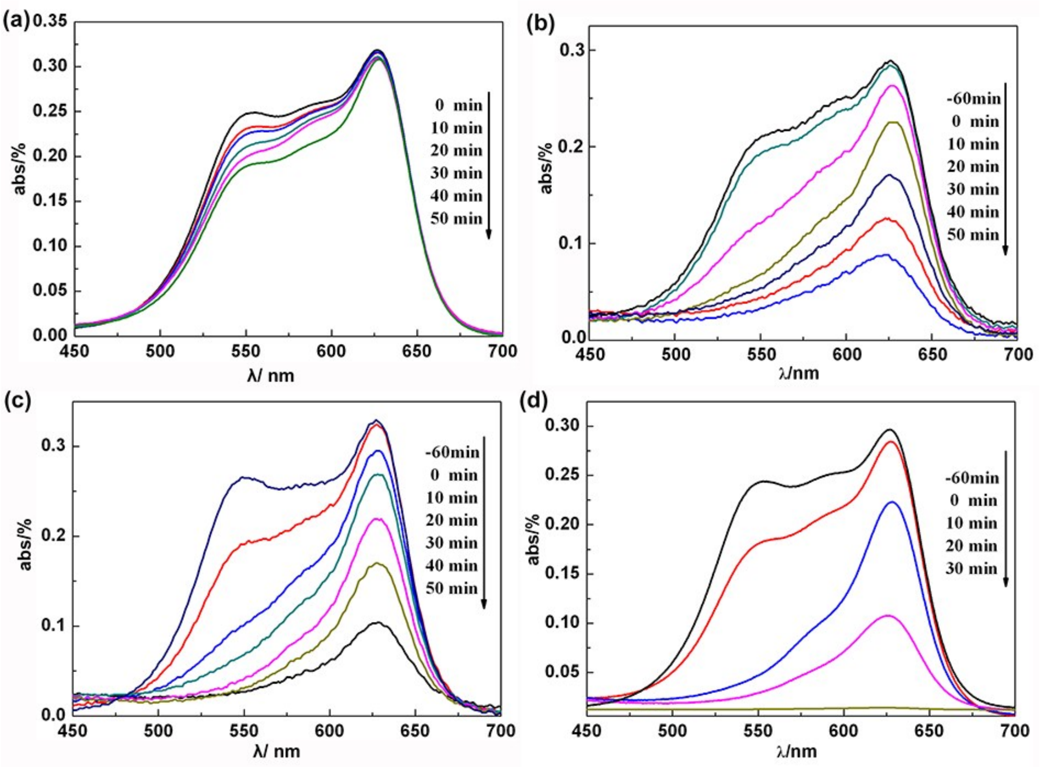

Fig. S1. The time-dependent absorption spectra of MB solution in the presence of (a) blank, (b) HZO (unanneal), (c) HZO (anneal) and (d) HZO@Gr.
